# Conventional and digital Ki67 evaluation and their correlation with molecular prognosis and morphological parameters in luminal breast cancer

**DOI:** 10.1038/s41598-022-11411-5

**Published:** 2022-05-17

**Authors:** Laura Pons, Laura Hernández-León, Ahmad Altaleb, Esperança Ussene, Roman Iglesias, Ana Castillo, Paula Rodríguez-Martínez, Eva Castella, Vanesa Quiroga, Eudald Felip, Beatriz Cirauqui, Mireia Margelí, Pedro Luis Fernández

**Affiliations:** 1 Department of Pathology, Hospital Germans Trias i Pujol, IGTP, Badalona, Spain; 2 Histopathology Department, Mubarak Al-Kabeer Hospital, Jabriya, Kuwait; 3 Department of Pathology, Hospital Vila Franca de Xira, Vila Franca de Xira, Portugal; 4 Department of Pathology, Hospital Santa Barbara, Puertollano, Ciudad Real Spain; 5 Medical Oncology Department, Catalan Institute of Oncology, B-ARGO Groups, IGTP, Badalona, Spain; 6 Department of Pathology, Hospital Germans Trias i Pujol, IGTP, Universitat Autònoma de Barcelona, Badalona, Barcelona Spain

**Keywords:** Cancer, Biomarkers, Oncology

## Abstract

Digital counting methods were developed to decrease the high intra- and inter-observer variability of immunohistochemical markers such as Ki67, with most presenting a good correlation coefficient (CC). Since Ki67 is one of the major contributors to Oncotype DX, it is conceivable that Ki67 expression and the recurrence score (RS) obtained by the multigene panel are positively correlated. We decided first to test to what extent conventional and digital Ki67 quantification methods correlate in daily practice and, second, to determine which of these methods correlates better with the prognostic capacity of the Oncotype DX test. Both Ki67 evaluations were performed in 89 core biopsies with a diagnosis of estrogen receptor (ER) positive HER2-negative breast cancer (BC). Cases were, thus, classified twice for surrogate subtype: first by conventional analysis and then by digital evaluation. The Oncotype RS was obtained in 55 cases that were subsequently correlated to Ki67 evaluation by both methods. Conventional and digital Ki67 evaluation showed good concordance and correlation (CC = 0.81 (95% CI 0.73–0.89)). The correlation of Oncotype DX risk groups and surrogate derived subtypes was slightly higher for the digital technique (r_s_ = 0.46, p < 0.01) compared to the conventional method (r_s_ = 0.39, p < 0.01), even though both were statistically significant. In conclusion, we show that digital evaluation could be an alternative to conventional counting, and also has advantages for predicting the risk established by the Oncotype DX test in ER-positive BC. This study also supports the importance of an accurate Ki67 analysis which can influence the decision to submit ER-positive HER2-negative BC to prognostic molecular platforms.

## Introduction

The pioneering studies conducted with microarrays for gene expression profiling performed by Sørlie et al. and Perou et al. generated a molecular portrait for classifying breast cancers (BC) into four intrinsic subtypes with different clinical outcomes, i.e., luminal A and B, HER2-positive and basal-like tumors^1,2^.

Based on an immunohistochemical (IHC) expression panel of estrogen receptors (ER), progesterone receptors (PR), HER2 and Ki67, BC have been classified in four surrogate subtypes: triple negative, HER2-positive and two types of luminal, the latter being the most frequent. Although the differentiation between these subtypes has generated much controversy, the 2017 St. Gallen consensus agreed that Ki67 and progesterone expression could be used to distinguish between luminal-A and luminal B-like^3^.

Ki67 IHC expression has been proposed as an independent prognostic factor in BC^4–10^. Its usefulness has been demonstrated by several trials, including the results from the Breast International Group Trial 1–98, whose work confirmed that higher values of Ki67 were associated with worse disease-free survival and with adverse prognostic factors^11–13^. Despite the publication of some guidelines^9,14^, there is no clear consensus regarding the criteria for its evaluation: number of cells to count, number of areas to select in heterogeneous tumors or the cut-off point^15^. In addition, the lack of a highly analytically validated assay and scoring system has led to a limited clinical utility. For this reason, the International Ki67 in Breast Cancer Working Group recently agreed that Ki67 IHC could be used in the decision-making treatment only if the results are below 5% and above 30%^16,17^. In an attempt to reduce the effect of all these variables, digital counting methods for this and other IHC biomarkers have been developed, with most presenting good correlation coefficients^18,19^. However, it is generally considered preferable to analyze gene expression signatures for prognostic purposes in luminal cancers when affordable and adequate reproducibility is not guaranteed^3^.

Several prognostic molecular signatures for BC are currently available, such as Oncotype Dx, Prosigna, MammaPrint and others. Oncotype DX is used not only as a prognostic factor in ER-positive, HER2-negative BC, but according to the results of the TAILORx trial, it is also a predictor of the benefits of chemotherapy in node negative patients^20–23^. More recently, the RxPONDER trial demonstrated that adjuvant therapy could be de-escalated to endocrine therapy alone in postmenopausal patients with a recurrence score (RS) ≤ 25 and 1 to 3 positive lymph nodes^23^. This molecular signature is based on the study of a total of 21 genes (16 cancer-related genes and 5 reference genes) and provides a RS from 0 to 100. This score initially sub-classifies patients into three risk groups (RG): low (RS = 0–17), intermediate (RS = 18–30) and high (RS = 31–100)^9,24^. The TAILORx trial then validated its clinical utility in node negative ER-positive HER2-negative BC, classifying patients into three risk groups according to different cut-offs: low (RS = 0–11), intermediate (RS = 12–25) and high (RS = 26–100)^20^.

As mentioned previously, a Ki67 percentage > 5 and < 30 causes uncertainty and should not be used in the decision-making treatment. In these cases, the decision to perform a gene-expression molecular profile is indicated. On the other hand, since Ki67 is one of the major contributors to the multigene panel^25^, it is conceivable that Ki67 expression and the Oncotype DX RS are positively correlated, as it was showed by Phase III PlanB Trial^26^. Taking all this into account, we decided to first test to what extent the conventional and digital Ki67 quantification methods correlate in daily practice and, second, to determine which of these methods correlates better with the prognostic information provided by the Oncotype DX test.

## Materials and methods

### Patients

Eighty-nine needle core biopsies corresponding to patients with a diagnosis of ER-positive HER2-negative invasive BC were selected from the pathology files of our hospital in 2019. All tumors were ER-positive and HER2-negative. Case selection was irrespective of tumor grade, size or age at diagnosis. All cases were treatment-naïve and obtained from the biobank of our institution. ER and PR were considered negative if IHC expression was < 1%. The clinicopathological characteristics of these 89 cases were obtained from the pathology reports and are shown in Table 1.

**Table 1:** Clinicopathological characteristics.

Variables	N	%
Patients	89	100
Age (mean ± SD)	63.03 ± 13.28	
**Tumor subtype**		
No special type	69	77.53
Lobular	8	8.99
Others (mucinous, tubular, mixed)	12	13.48
**Histological grade**		
Grade 1	27	30.34
Grade 2	55	61.79
Grade 3	7	7.87
**Tubule formation**		
Score 1: > 75%	4	4.50
Score 2: 10–75%	20	22.47
Score 3: < 10%	57	64.04
Unknown	8	8.99
**Nuclear pleomorphism**		
Score 1	14	15.73
Score 2	52	58.43
Score 3	15	16.85
Unknown	8	8.99
**Mitotic counts**		
Score 1	65	73.03
Score 2	12	13.48
Score 3	4	4.50
Unknown	8	8.99
**ER status**		
Positive	89	100
Negative	0	0
Unknown	0	0
**PR status**		
Positive	84	94.38
Negative	5	5.62
Unknown	0	0
**pT stage**		
T1a	0	0
T1b	19	21.35
T1c	33	37.08
T2	33	37.08
T3	4	4.50
T4	0	0
**pN stage**		
N0	77	86.52
N1	12	13.48

All patients provided informed consent for this study, which was evaluated and approved by the Institutional Research Board of our institution. All methods were performed in accordance with the relevant guidelines and regulations.

### Tissue preparation and immunohistochemistry

Needle biopsies were processed following current ASCO/CAP guidelines for optimal tissue handling. Three-micrometer-thick sections were obtained from the blocks and subjected to heat-induced antigen retrieval. Immunochemical staining of Ki67 was performed using a prediluted rabbit monoclonal antibody against human Ki67 (Clone 30-9, Ventana, Tucson, AZ, USA) and carried out in a Benchmark Ultra stainer using an UltraView detection kit. A similar procedure was also used for estrogen (clone SP1) and progesterone receptors (clone 1E2) and HER2 (clone 4B5).

### Conventional and digital Ki67 quantification

Following the same methodology, the two expert pathologists who signed out the cases, also performed conventional Ki67 quantification and the mean was calculated. To decrease interobserver variability, a consensus was reached regarding the evaluation method: in cases with homogeneous staining, at least two randomly selected high-power fields were evaluated. In cases with a heterogeneous distribution of Ki67, one area with the highest expression (hot spot) and one area with the lowest expression (cold spot) were selected. The average of the two areas was calculated for the final Ki67 assessment. A minimum of 200 cells/area was counted in all cases.

**Figure 1:**
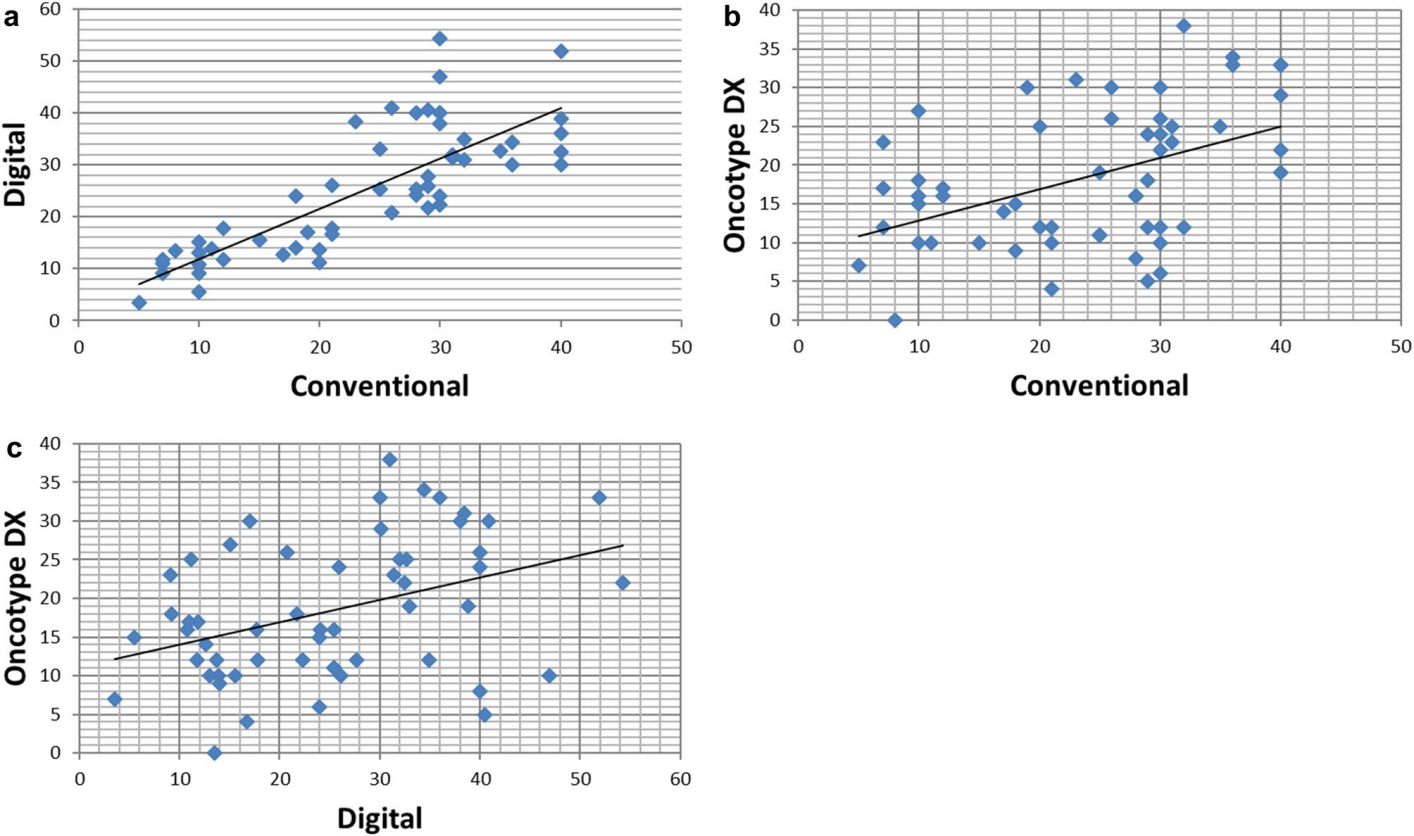
Dispersion of Ki67 quantification by conventional and digital methods showing a strong and linear positive correlation (**A**), and dispersion of the Oncotype DX Recurrence Score with Ki67 quantification by conventional (**B**) and digital (**C**) evaluation, showing a moderate and linear positive correlation.

Digital images were captured using the Roche Ventana iScan HT slide scanner with an × 20 objective. Automated scanning processes (placement of focus points, selection of scanning area) were checked by a laboratory technician and repeated when necessary. Images were saved as bif files. Semiautomated digital analysis was performed with the Virtuoso software (Ventana) as shown in Fig. 1. A pathologist performed the area selection with the same criteria as that used for conventional evaluation. Areas with ductal carcinoma in situ were excluded manually from automated scoring. A minimum of 200 cells was automatically evaluated in all cases.

### Ki67 cut-off point

Ki67 quantification has been proposed for surrogate subtype classification in ER-positive HER2-negative BC. Since there is no consensus regarding an adequate cutoff-point, calculating the institutional mean has been recommended^12^. Ki67 values from all the core biopsies diagnosed as infiltrating BC within 1 year in our department generated a mean of 23.7. Thus, in this study, we defined “Luminal A-like” as ER-positive, HER2-negative and Ki67 ≤ 24%, and “Luminal B-like” as ER-positive, HER2-negative and Ki67 > 24%.

Thus, cases were classified twice for surrogate subtype; first with the conventional Ki67 evaluation method and, second, with digital evaluation.

### Oncotype DX recurrence score (RS)

Oncotype DX was analysed on surgical specimens. The RS was obtained in 55 cases which were subsequently classified into low (RS = 0–17), intermediate (RS = 18–30) and high risk (RS = 31–100)^9,24^. The decision to perform this test was based on the recommendations of the clinical guidelines of our center, [ICOPraxis^27^], which takes into account several parameters including tumor grade, hormonal receptors and Ki67 expression. The RS obtained in each case was correlated with pathologic variables using the Pearson correlation coefficient (r) for continuous variables (digital and conventional Ki67 quantification) and the Spearman’s rank order correlation coefficient (r_s_) for ordinal variables (Ki67-derived sub-classification, histological grade, tubule formation, nuclear pleomorphism and mitotic score), with a two-tailed p-value of < 0.05 being considered as significant.

## Results

Table 1 shows the distribution of clinicopathological variables of the cases analysed. We first compared the results of Ki67 evaluation by both the conventional and the digital method.

The concordance between the two pathologists, from whose evaluation the Ki67 mean was obtained, was good (concordance: 0.89, kappa index: 0.79). Also, a good intraclass correlation coefficient of 0.81 (95%confidence interval [CI] 0.73–0.89) between conventional and digital methods was obtained (Fig. 1A).

When a Ki67 cut-off point of 24% was used to classify these cases into surrogate subtypes (luminal A-like and luminal B-like), the categorical concordance between the two methods could be calculated. In our study, 43 cases were initially classified as luminal A-like and 46 as luminal B-like with the conventional Ki67 evaluation technique, whereas 44 cases were classified as luminal A-like and 45 as luminal B-like with the digital method. This means that both Ki67 evaluation methods have a very good correlation for establishing the tumor luminal subtype (concordance rate of 0.89; kappa index 0.786). There were, however, 9 discordant cases in the surrogate subtype classification indicating that they may have non-coincidental results in individual cases which might have clinical consequences.

We then sought to establish which of these Ki67 evaluation methods correlated better with the prognostic information generated by the Oncotype DX test and found that although both were significantly correlated (Fig. 1B,C), conventional Ki67 quantification showed higher concordance with the Oncotype RS (R = 0.45, p < 0.001) than the digital technique (R = 0.39, p = 0.003).

**Figure 2:**
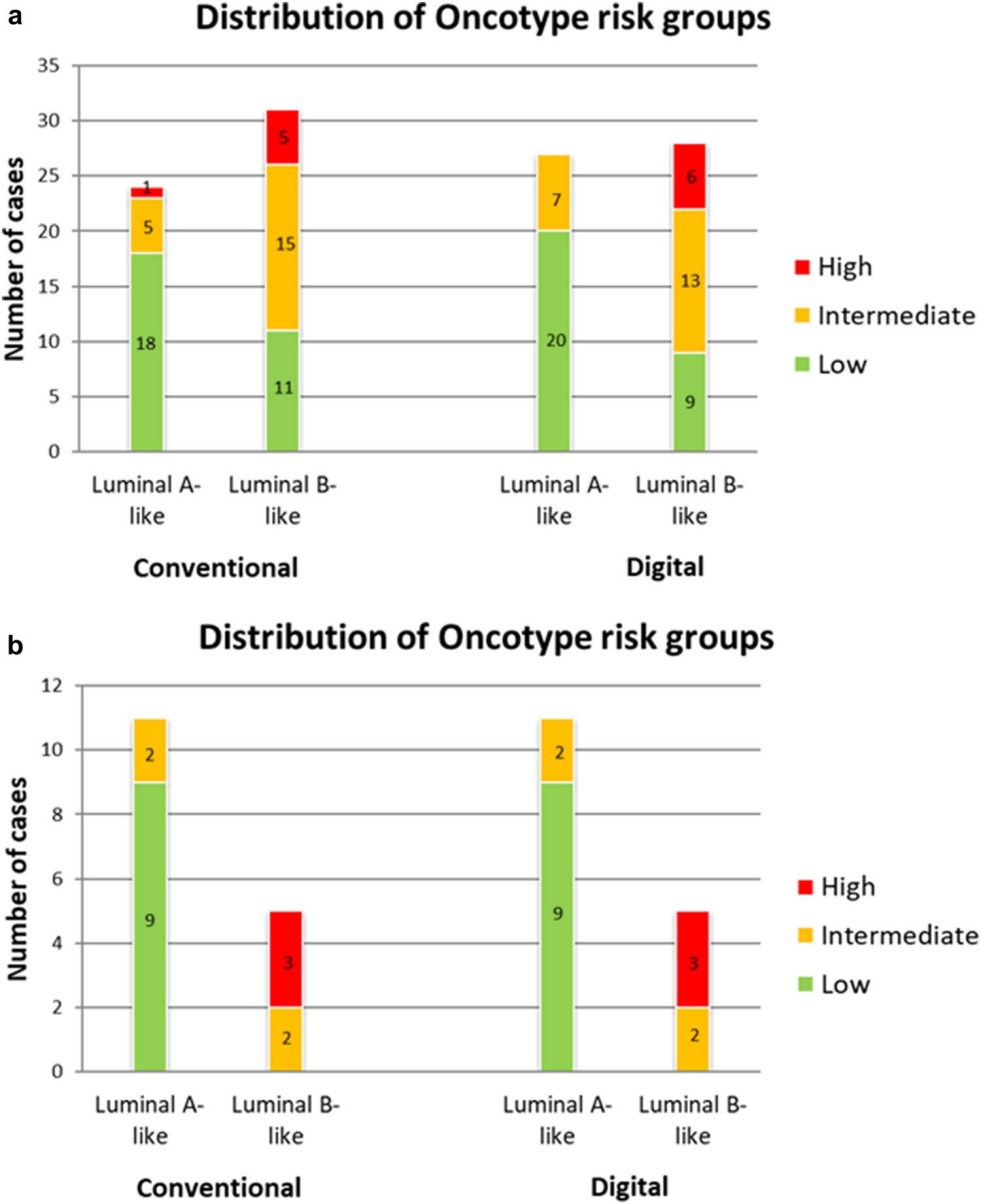
Distribution of the Oncotype DX risk groups within the surrogate subtypes derived from conventional and digital methods of Ki67 quantification, showing the classification into surrogate subtypes by conventional and digital methods using Ki67 mean as cut-off point (**A**) and Ki67 mean-SD and mean + SD discarding the grey zone (**B**).

We then compared the surrogate tumor subtype classification derived from both methods with the risk groups derived from Oncotype DX. In our analysis of 55 cases, 29 had been classified into the low risk, 20 into the intermediate risk and 6 into the high-risk groups. The distribution of the risk groups (RG) and surrogate subtypes derived from both counting methods is shown in Fig. 2A.

The conventional method classified 24 tumors as luminal A-like and 31 as luminal B-like. In contrast, 27 tumors were classified as luminal A-like and 28 as luminal B-like by the digital method. The distribution of the high RG (RS = 31–100) by both methods varied in one case only, which was classified as luminal A-like by the conventional method and upgraded to luminal B-like by the digital method. The distribution of the low RG (RS = 0–17) was also very similar, with only two discordant cases classified as luminal B-like by the conventional method, but downgraded to luminal A-like by the digital technique. The intermediate RG (RS = 18–30) also showed discordance in two cases, which were classified as luminal B-like according to the conventional method and downgraded to luminal A-like by the digital method. The correlation of these categorical variables (Oncotype DX RG and surrogate-derived subtypes) was statistically significant for the digital (r_s_ = 0.46, p < 0.01), as well as for the conventional technique (r_s_ = 0.39, p < 0.01). We then tried to improve the results by using three intervals considering the median-SD and median + SD and discarding those cases in the middle grey zone (> 14 y < 34). This caused a n important reduction in the subjects of the sample. Our results showed that there were no differences in the distribution of Oncotype RG between conventional and digital methods (Fig. 2B). The only difference was that there was no cases previously considered luminal A-like by conventional method and classified as high risk by Oncotype DX.

The correlation of Oncotype DX with other pathologic variables is described in Tables 2 and 3. The Nottingham grade and mitotic score were significantly correlated with the Oncotype DX RG (r_s_ = 0.38, p < 0.01; r_s_ = 0.43, p < 0.01; respectively). Although tubule formation and nuclear pleomorphism are two of the three contributors to the Nottingham grade, they were not significantly correlated with the Oncotype DX RG (r_s_ = 0.22, p = 0.11; r_s_ = 0.19, p = 0.17; respectively).

**Table 2:** Frequencies of clinicopathological parameters for Oncotype DX risk group’s categories.

N = 55	Oncotype DX risk group		
	Low (n = 29)	Intermediate (n = 20)	High (n = 6)
**Histological grade**			
G1	11 (37.93%)	3 (15%)	0
G2	18 (62.07%)	15 (75%)	5 (83.33%)
G3	0	2 (10%)	1 (16.67%)
**Tubular formation**			
Score 1	2 (6.89%)	0	0
Score 2	8 (27.59%)	5 (25%)	0
Score 3	19 (65.52%)	15 (75%)	6 (100%)
**Nuclear pleomorphism**			
Score 1	7 (24.14%)	2 (10%)	1 (16.67%)
Score 2	19 (65.52%)	13 (65%)	4 (66.67%)
Score 3	3 (10.34%)	5 (25%)	1 (16.67%)
**Mitotic counts**			
Score 1	28 (96.55%)	14 (70%)	3 (50%)
Score 2	1(3.45%)	4 (20%)	3 (50%)
Score 3	0	2 (10%)	0

**Table 3:** Correlation of variables with Oncotype Dx test, showing the test used in each parameter, its value and the significance (p).

	Test	Value	p
Ki67 conventional quantification	Pearson (R)	0.45	< 0.001
Ki67 digital quantification	Pearson (R)	0.39	0.003
Ki67 conventional—derived subtype	Spearman (r_s_)	0.39	0.003
Ki67 digital—derived subtype	Spearman (r_s_)	0.46	< 0.001
Histological Grade	Spearman (r_s_)	0.37	0.004
Tubule formation	Spearman (r_s_)	0.22	0.101
Nuclear pleomorphism	Spearman (r_s_)	0.19	0.169
Mitotic counts	Spearman (r_s_)	0.43	0.001

### Ethical approval

We confirm that Ethical Committee from Hospital Universitari Germans Trias I Pujol approval was sought for this project. Written informed consent for genetic platform and for specifical project was obtained for participants.

## Discussion

Our analysis of 89 ER-positive HER2-negative BC, showed good concordance of conventional and digital methods of Ki67 expressions. This is in line with previous publications, which also obtained high levels of correlation^19,28^. A good intraclass correlation coefficient was obtained, suggesting that both methods might be equivalent for routine assessment of this biomarker. Given this correlation, the possibility of the digital method definitively replacing conventional counting seems at hand, at a time in which digital pathology has increasing importance. This would decrease the interobserver variability by using an almost automated nuclear counting as well as the time spent on cumbersome microscopic evaluation. Nevertheless, automation should not preclude a very recommendable institutional validation before being implemented.

After having demonstrated the concordance of the two methods, we decided to determine which Ki67 evaluation technique correlated better with the prognostic capacity of Oncotype DX, a test frequently used to classify the risk of luminal BCs in order to make therapeutical decisions regarding the addition of adjuvant chemotherapy to hormonal treatment. There was a statistically significant correlation between the RS and Ki67 quantification (continuous variables) with both methods, being the conventional slightly higher than the digital method. This demonstrates that Ki67 evaluation provides relevant prognostic information although, apparently, traditional microscopic evaluation might have some advantages in spite of its inherent variability. Nevertheless, this correlation was not perfect, which most likely indicates that while proliferation is important in the profile analysed, it is not the only parameter measured by the molecular Oncotype DX test. This is in line with the study conducted by Paik et al., which showed similar correlation between Ki67 average labeling index and Oncotype DX RS (r = 0.52, p < 0.0001)^29^.

The correlation between surrogate subtypes derived from the Ki67 quantification and the Oncotype RG (categorical variables) also showed good concordance, being the digital a better method in this case, since it selected all high risk and less low risk cases. Indeed, if the histopathological phenotypic classification is based on Ki67 immunohistochemical expression, among other parameters, and if risk categorization by Oncotype DX were considered a “gold standard” or good reference, conventional Ki67 assessment would have classified 1 case of luminal A-like BC in the high-risk group, whereas digital evaluation would have classified only luminal B cases as high risk. Similarly, all cases classified as high risk by Oncotype DX would fall in the luminal B group defined by the digital method. However, the limited number of cases in this high RG does not allow definitive conclusions to be drawn in this regard and larger series are needed.

Our data also showed that 95% of the cases with a Ki67 ≥ 30% obtained by the digital method were in the high RS group, whereas only 90% of the cases with the same Ki67 value were in the high group by the conventional method. This suggests that the digital method could have advantages for defining a more appropriate Ki67 range in which it is recommended to perform a gene platform.

To our knowledge, this is the first study correlating both Ki67 evaluation methods with what is currently considered one of the gold standard molecular techniques for predicting the prognosis of women with ER-positive HER2-negative BC. Our results therefore support the use of digital Ki67 evaluation as an alternative method to classical cell counting while also providing some advantages for assessing the risk by more precisely labeling cases as luminal B-like.

Lastly, we attempted to establish the possible relationship between histopatopathological parameters and the information provided by the Oncotype DX test in luminal BC. The correlation of Oncotype DX RS with the Nottingham grade was studied in previous publications^30,31^, and showed a statistically significant correlation, being the high RS associated with a high histological grade. Another study also concluded that the Oncotype DX result is similarly impacted by histologic grade^32^. We also found this positive correlation, as well as with other histopathological variables such as mitotic counts, which, not surprisingly, showed the strongest correlation with Oncotype DX. This highlights the importance of reporting these parameters since they can be useful for decision making in cases for which Oncotype DX or other molecular tests are not feasible.

We found no statistically significant correlation between Oncotype DX and nuclear pleomorphism or tubule formation, despite both items being assessed in the Nottingham grade. This suggests that the correlation between this grading system and Oncotype DX derives from the correlation of Ki67, the expression of which is evaluated by both the histological grade and Oncotype DX. The reason for the lack of correlation between the molecular test and both pathological parameters (nuclear pleomorphism and tubular formation) might be that the latter are more subjective variables than mitotic index and the Oncotype DX is based on the expression of 21 genes including Ki67, ESR1 and HER2, which do not perfectly correlate with morphological parameters. Nevertheless, and despite not being statistically significant, we observed an interesting relationship between tubule formation and the Oncotype DX RG. Indeed, as seen in Table 2, 100% of high-risk cases had a score of 3 in tubule formation, which could indicate that poor tubule formation is strongly suggestive of a poor prognosis and this parameter could help to recommend the molecular test in luminal BC, if validated in larger series of cases.

One of the limitations of the study is the fact that comparing Ki67 index from core biopsy and Oncotype DX performed in whole section may influence our results due to the possibility of a heterogeneous distribution of Ki67 expression and the difference of amount of tissue evaluated with each type of sample. We have consider such possibility but the current literature shows contradictory results. On one hand, the study conducted by Ahn et al. proved significant differences in the Ki67 index between core biopsy and surgical specimens^33^, in contrast with other work^34^ which shows significantly concordant results. Nevertheless, a large recent study which included more than 4000 patients and evaluated the differences in the prognostic value of Ki67 between core biopsy and postoperative specimen, concludes that Ki67 is an important prognostic factor regardless of the timing of testing, although it is recommended to analyse this parameter preoperatively^35^. In our study, we decided to use only Ki67 score from biopsies in order to maintain the routine procedure and thus increase the clinical value of our analysis, although acknowledging the above-mentioned bias.

The main limitation of our study is the size of our series that precludes absolutely definitive conclusions. Another limitation of this study is that the correlation between Ki67 evaluation and Oncotype results was based on the mean of two quantifications of the former, whereas in common practice it is only one pathologist who provides this result, and this could influence the analysis. Nevertheless, our work is unique in having a correlation between Ki67 classical and digital scoring, surrogate subtypes and results from Oncotype Dx.

In conclusion, we demonstrate that conventional and digital methods for Ki67 evaluation correlate well and that digital evaluation could be an alternative to conventional counting and can also provide advantages when making the decision to indicate the molecular test Oncotype DX in cases with a Ki67 index over 30%. This study also confirms the importance of an accurate Ki67 evaluation method which can influence the decision to submit ER-positive HER2-negative BC to prognostic molecular platforms as well as the possible usefulness of classical parameters such as tubule formation which can provide additional complementary information t for decision making.

## Data Availability

The datasets generated and/or analysed during the current study are not publicly available, but are available from the corresponding author on reasonable request. The policy of Hospital Universitari Germans Trias i Pujol (HUGTiP) is to share with the scientific community any data obtained in research projects, as long as ethical and legal regulations permit it. Our institution strives to publish the results, as well as supporting data in its raw, processed and analyzed states, in a long-term data archive to which access may be open, or restricted, or both. HUGTiP recommends that while research is ongoing, data is stored on the institute server. For this purpose, our group has its own server space which is supported by the IT department. This server space allows for managed access to and the sharing of data between and among partners during the project. Safe and secure storage is guaranteed by the IT security and safety protocols of the institute network. If it is not possible to store the data directly on the institute network, data stored is encrypted on a local device (laptop) and transferred to the institute network as soon as possible. HUGTiP has set strict conditions for the management of research data. In accordance with the Institute’s research data management policy all research data will be archived permanently for scientific integrity reasons. All data suitable for reuse will be made available to the scientific community, together with their accompanying metadata and documentation necessary to understand the data. To this end, services of the Research Information System of HUGTiP will be used. Via RIS, data sets are made available, a long-term data archive to which access may be open, or restricted, or both.
